# Comparative Transcriptome Analysis of *Litopenaeus vannamei* Reveals That Triosephosphate Isomerase-Like Genes Play an Important Role During Decapod Iridescent Virus 1 Infection

**DOI:** 10.3389/fimmu.2020.01904

**Published:** 2020-08-28

**Authors:** Xuzheng Liao, Chenggui Wang, Bo Wang, Haipeng Qin, Shikang Hu, Ping Wang, Chengbo Sun, Shuang Zhang

**Affiliations:** ^1^College of Fisheries, Guangdong Ocean University, Zhanjiang, China; ^2^Hainan Zhongzheng Aquatic Science and Technology Co., Ltd., Hainan, China; ^3^Guangdong Provincial Laboratory of Southern Marine Science and Engineering, Zhanjiang, China; ^4^Guangdong Provincial Key Laboratory of Pathogenic Biology and Epidemiology for Aquatic Economic Animals, Zhanjiang, China; ^5^Aquatic Animals Precision Nutrition and High Efficiency Feed Engineering Research Center of Guangdong Province, Zhanjiang, China

**Keywords:** *Litopenaeus vannamei*, DIV1, triosephosphate isomerase, transcriptome analysis, RNA interference

## Abstract

Decapod iridescent virus 1 (DIV1) results in severe economic losses in shrimp aquaculture. However, little is known about the physiological effect of DIV1 infection on the host. In this study, we found that the lethal dose 50 of DIV1-infected *Litopenaeus vannamei* after 48, 72, 96, and 156 h were 4.86 × 10^6^, 5.07 × 10^5^, 2.13 × 10^5^, and 2.38 × 10^4^ copies/μg DNA, respectively. In order to investigate the mechanisms of DIV1 infection, a comparative transcriptome analysis of hemocytes from *L. vannamei*, infected or not with DIV1, was conducted. The BUSCO analysis showed that the transcriptome was with high completeness (complete single-copy BUSCOs: 57.3%, complete duplicated BUSCOs: 41.1%, fragmentation: 0.8%, missing: 0.8%). A total of 168,854 unigenes were assembled, with an average length of 601 bp. Based on homology searches, Kyoto Encyclopedia of Genes and Genomes (KEGG), gene ontology (GO), and cluster of orthologous groups of proteins (KOG) analysis, 62,270 (36.88%) unigenes were annotated. Among them, 1,112 differentially expressed genes (DEGs) were identified, of which 889 genes were up-regulated and 223 genes were down-regulated after DIV1 infection. These genes were mainly annotated to the major metabolic processes such as fructose and mannose metabolism, carbon metabolism, and inositol phosphate metabolism. Among these metabolic pathways, the triosephosphate isomerase (*TPI*) family was the most eye-catching DEG as it participates in several metabolic processes. Three types of *TPI, LvTPI-like, LvTPI-Blike*, and *LvTPI-Blike1*, were obtained for gene silencing by RNA interference. The results showed that *LvTPI-like* and *LvTPI-Blike1* silencing caused a high mortality rate among *L. vannamei*. However, *LvTPI-like* and *LvTPI-Blike* silencing reduced DIV1 replication in DIV1-infected *L. vannamei*. All the results indicated that *TPI-like* genes play an important role during DIV1 infection, which provides valuable insight into the infection mechanism of DIV1 in shrimp and may aid in preventing viral diseases in shrimp culture.

## Introduction

*Litopenaeus vannamei* is a widely cultured shrimp species all around the world, with a huge production per year (1). The development of farmed shrimp has led to high-density growth conditions, large-scale production, and unsanitary aquaculture wastewater discharge, resulting in disease overflow, ecological imbalance, and environmental deterioration. The shrimp industry is now faced with finding solutions for these serious problems (2, 3). Over the past decades, diseases caused by various bacterial, fungal, parasitic, and viral species have significantly constrained the productivity of the *L. vannamei* industry (4). For a long time, the most concerning viruses were the white spot syndrome virus (WSSV), the Taura syndrome virus (TSV), and the infectious hypodermal and hematopoietic necrosis virus (IHHNV) (5). However, in 2014, the decapod iridescent virus 1 (DIV1) caused huge losses in farmed *L. vannamei* in Zhejiang Province in China. DIV1 was isolated and identified by Qiu et al. in 2017 (6). Since then, the prevention and the control of DIV1 have attracted much attention in shrimp culture.

In 1993, Lightner and Redman first discovered the iridescent virus in shrimp in Ecuador (7). In 2004, Tang et al. found the iridescent virus in *Acetes erythraeus* grown in Madagascar and, *via* sequencing, found that it was a new type of iridescent virus (*Sergestid iridovirus*, SIV) (8). In 2016, Xu et al. detected a new iridescent virus, *Cherax quadricarinatus* iridovirus (CQIV), from *Cherax quadricarinatus* on a farm in China (9). In 2017, Qiu et al. detected shrimp hemocyte iridescent virus (SHIV) from *L. vannamei* and determined that SHIV is a member of the new genus *Xiairidovirus*, which also belongs to the Iridoviridae family. The complete genome sequence of SHIV is 165,908-bp long with 34.6% G + C content and 170 open reading frames. Qiu et al. used intermuscular injection and reverse gavage methods to infect *L. vannamei* with SHIV, resulting in a 100% cumulative mortality rate. Results from the histopathological study using transmission electron microscopy of ultrathin sections and *in situ* hybridization indicated that SHIV mainly infects the hematopoietic tissue and hemocytes in the Pacific white shrimp (6). In 2019, the Executive Committee of the International Committee on Taxonomy of Viruses (ICTV) identified two virus isolates, SHIV and CQIV, as decapod iridescent virus 1 (DIV1) (10). Crustaceans in the coastal region of China, including *L. vannamei, Fenneropenaeus chinensis, Exopalaemon carinicauda*, and *Macrobrachium rosenbergii*, can all carry DIV1 (6, 11, 12). Until now, most of the studies on DIV1 focused on the virus itself or the histopathological changes in the host. Latest studies in 2020 based on transcriptome analysis showed that the phagosome and the MAPK signaling pathway were positively modified during DIV1 infection in *C. quadricarinatus* (13), while lysosome and phagosome were induced during DIV1 infection in *Fenneropenaeus merguiensis* (14). However, little is known about the mechanism of the host response to DIV1 infection.

Shrimp rely on their innate immune system to defend against invading viruses and microbes. Shrimp cells can recognize the invading virus *via* unique host pattern recognition proteins with pathogen-associated molecular patterns, which can activate the host immune response (15). The innate immune system includes the humoral immune system and the cellular immune system. The humoral responses are mediated by macromolecules in the hemolymph. Humoral responses are mainly divided into melanin synthesis by the prophenoloxidase system, the blood clotting system, and the generation of circulating antimicrobial peptides (16). The cellular immune response involves different types of hemocytes, which clear harmful substances in the hemolymph by defensive reactions such as phagocytosis and encapsulation (17). Recent studies confirmed that hemocytes are an important source of several humoral effector molecules, which are required in killing foreign invaders in shrimp (18, 19). It is necessary to understand the immune system of shrimp in order to develop methods that can successfully control and reduce the loss of shrimp production due to infectious diseases. High-throughput RNA sequencing (RNA-Seq) is an efficient technology to analyze gene expression, discover transcripts, and select differentially expressed genes (DEGs) (20). This technology has been used to study the molecular basis of certain gene transcription processes (21). Ren et al. found several genes related to immunity through the transcriptome profiles of *M. japonicus* following infection with *V. parahemolyticus* or WSSV (22). Additional research into the function of these immune genes, such as *caspase 4, integrin, crustin, ubiquitin-conjugating enzyme E2, C-type lectin*, and α_2_*-macroglobulin*, is required to understand the molecular interactions between *V. parahemolyticus* and WSSV in *M. japonicus* and to provide valuable information for preventing diseases (22). However, no information is available on the gene expression profiles of DIV1-infected *L. vannamei*.

In the present study, the lethal concentration 50 (LD_50_) of DIV1-infected *L. vannamei* was determined, and RNA-Seq was applied to compare the transcriptome difference between the DIV1-infected and non-infected *L. vannamei*. This study aims to gain a better insight into the DIV1–shrimp interaction and may help better understand the innate immune mechanism in shrimp, which would be beneficial to disease prevention in shrimp culture.

## Materials and Methods

### Shrimp Culture

The study protocol was approved by the ethics review board of the Institutional Animal Care and Use Committee in Guangdong Ocean University. *L. vannamei* (body weight 11.2 ± 2.4 g) was purchased from Hainan Zhongzheng Aquatic Science and Technology Co., Ltd., in Dongfang (Hainan, China). The shrimps were acclimatized for 1 week in 0.3-m^3^ tanks with aerated and filtered seawater in East Island Marine Biological Research Base, Guangdong Ocean University in Zhanjiang, Guangdong, China. The holding seawater conditions were as follows: salinity at 28.5 ± 0.26 %0, pH at 8.17 ± 0.01, and temperature at 29.3 ± 0.5°C. Commercial feed was used to feed the shrimp three times a day. The shrimps were then randomly sampled and tested by PCR to ensure that they were free from WSSV, IHHNV, and DIV1 using the primers shown in Table S1.

### LD_50_ Test

DIV1 was obtained from a Peihua prawn farm in Wuchuan, Guangdong, China, and the virus was extracted from the infected tissue of *L. vannamei*, as conducted previously (23). The DIV1 inoculation was tested by PCR to ensure that it was not contaminated with the DNA of any other known crustacean virus (e.g., WSSV and IHHNV). DNA was extracted using the EasyPure Marine Animal Genomic DNA Kit (Transgen, Beijing, China). Extracted DNA was quantified using SimpliNano (GE Healthcare, US). The DNA samples of the pleopods were used to detect the viral loads by real-time PCR performed in a LightCycler (Roche) with the following program: denaturation at 95°C for 30 s, followed by 40 cycles at 95°C for 5 s and 60°C for 30 s, using the primers qRT-DIV1-F, qRT-DIV1-R, and Taqman Probe (Table S1) (24). Toxicity tests were performed with the same method as in the study of WSSV in shrimp (25). Six groups of healthy *L. vannamei* were intramuscularly injected at the third abdominal segment with 50 μl of DIV1 supernatants at five concentrations (2.14 × 10^8^, 2.14 × 10^7^, 2.14 × 10^6^, 2.14 × 10^5^, and 2.14 × 10^4^ copies/μg DNA) and phosphate-buffered saline (PBS; pH 7.4) as a control. Three replicates of 30 shrimps per replicate were used in each group. The conditions of the LD_50_ test were the same as discussed in “Shrimp Culture” section. The cumulative mortality was recorded every 4 h for the LD_50_ calculation. To investigate the copies of DIV1, total DNA was extracted from hemocyte, hepatopancreas, intestine, gill, and muscle of *L. vannamei* at 6, 12, 24, 48, and 72 h after DIV1 injection on the concentration at LD_50_ 48 h from infection.

### Transcriptome Sequencing and Analysis

#### Sample Collection

*L. vannamei* was intramuscularly injected with 50 μl of DIV1 supernatant based on LD_50_ 48 h after infection. *L. vannamei* injected with PBS was used as controls. At 48 h post-injection (hpi), the hemocytes from three shrimp were combined as one sample for transcriptome sequencing. The hemolymph was withdrawn into modified ACD anticoagulant solution, and the hemocytes were separated from plasma by centrifugation (3,000 × *g* for 5 min at 4°C) (26). The hemocytes of *L. vannamei* were immediately frozen in liquid nitrogen and stored at −80°C until RNA extraction. Three biological replicates were performed for the infection and the control groups, for a total of six samples. The extracted RNA was pooled for transcriptome sequencing.

#### RNA Extraction and Transcriptome Sequencing

Total RNA from the hemocytes of *L. vannamei* was isolated using TransZol Up Plus RNA Kit (Transgen, Beijing, China), and the RNA concentration was determined using SimpliNano (GE Healthcare, US). Fragmentation buffer was used to break the mRNA into short fragments. Using mRNA as a template, the first-strand cDNA strand was synthesized using random hexamers, followed by the addition of buffer, dNTPs, RNase H, and DNA polymerase I to synthesize the second-strand cDNA. Poly(A) was added to connect to the sequencing adaptor. Finally, the Illumina HiSeqTM platform was used to sequence the library at Guangzhou Sagene Biotech Co., Ltd. (Guangzhou, China).

#### *De novo* Assembly and Data Analysis

Raw reads were filtered to remove adaptor and low-quality sequences. After filtering, an RNA assembly of clean data from the mock and the DIV1-infected samples was performed with Trinity Assembly Software. The completeness of the assembly was assessed using BUSCO/v3.0.2 with the BUSCO arthropod dataset (27). Six functional databases were used to search for the unigenes, including NCBI protein NR (https://blast.ncbi.nlm.nih.gov/Blast.cgi), COG (https://www.ncbi.nlm.nih.gov/COG/), SWISS-PROT (https://www.expasy.org/), KEGG (https://www.genome.jp/kegg/), GO (http://geneontology.org/), and Pfam (http://asia.ensembl.org/index.html). In addition, Gene Ontology (GO) and metabolic pathway analysis were conducted using the Blast2GO program and KEGG program (https://www.genome.jp/kegg/), respectively.

#### Differential Expression Analysis and Functional Annotation

Log_2_(FC) was used as an indicator of the genetic transcriptome differences between the DIV1-infected and the control groups. Fragments per kilobase million was used as the measurement unit to estimate the expression level of each transcript in the study. False discovery rate (FDR) was also used to correct the calculated *p*-values (28). Genes with FDR ≤ 0.05 and |log_2_(FC)| >1 were considered to be DEGs. In addition, KEGG and GO were also used for DEGs pathway and GO enrichment analysis, respectively.

#### Validation of DEGs by qRT-PCR

To validate the transcriptome data, 2 μg of high-quality hemocyte RNA samples from the DIV1-infected group and the PBS control group was reverse-transcribed using the 5X All-in-One RT Master Mix (Applied Biological Materials, Vancouver, Canada) according to the manufacturer's protocol. The RNA concentration of the DIV1-infected group and the PBS control group was 120.48 and 156.64 μg/ml, respectively. A total of eight differentially expressed unigenes from the hemocyte transcriptome data of *L. vannamei* were selected for qPCR analysis to validate the transcriptome. All DEGs were validated by qPCR using a Light Cycler® 96 system (Roche Applied Science, Switzerland) in a final reaction volume of 20 μl, which was comprised of 2 μl of 1:10 cDNA diluted with ddH_2_O, 7.2 μl of ddH_2_O, 10 μl of TB Green Premix Ex Taq II (Takara Biomedical Technology, Beijing, China; Code No. RR420Q), and 10 μM of specific primers. The cycling program was as follows: 1 cycle at 95°C for 30 s, followed by 40 cycles at 95°C for 15 s, 62°C for 1 min, and 65°C for 15 s. Cycling ended at 95°C, with a 4.4°C/s calefactive velocity to create the melting curve. The primers used in the qPCR analysis are listed in Table S1. 2^−ΔΔ*Ct*^ method was used to calculate gene expression (29). The amplification efficiencies (*E*) were calculated using the formula provided by Bustin et al. (30). The expression level of each gene was normalized by *EF1*α (GenBank accession no. GU136229).

### Knockdown of *TPI* of *L. vannamei in vivo* Expression by Double-Stranded RNA-Mediated RNA Interference

Three types of triosephosphate isomerase (TPI)-specific primer sequences were linked to the T7 promoter by using the T7 RiboMAX™ Express Large Scale RNA Production System (Promega) to synthesize double-stranded RNAs (dsRNAs) following the method as previously described (31). The primers used for the synthesis of dsRNAs are shown in Table S1. The experimental group was injected with dsRNA-*LvTPI-likes* (2 μg/g), while the control groups were injected with equivalent dsRNA-EGFP. RNA interference efficiency was investigated using qPCR. The hemocyte samples were taken from nine shrimp in each challenge group at 24 and 48 hpi, and three shrimp were pooled together. Total RNA was extracted and reverse-transcribed into cDNA for qPCR. LvEF1-α was used as the internal control. The primer sequences are listed in Table S1.

### Bioassay of DIV1 and PBS Challenge Tests in *TPI*-Knockdown *L. vannamei*

Healthy *L. vannamei* (7.12 ± 1.05 g, *n* = 40) received an intramuscular injection of dsRNA-*LvTPI-like*, dsRNA-*LvTPI-Blike*, dsRNA-*LvTPI-Blike1*, dsRNA-EGFP, or PBS. dsRNA was injected at a concentration of 2 μg/g shrimp. Four replicates (three replicates for mortality calculation and one for the sample collection) were analyzed for each group. After 48 h, the shrimp were injected again with 2.01 × 10^4^ copies of DIV1 particles and mock-challenged with PBS as a control. The shrimp were cultured in tanks with air-pumped circulating seawater and were fed with artificial diet three times a day at 5% of body weight for about 7 days following the infection. The mortality of each group was counted every 4 h. At 24 and 48 h after DIV1 challenge, the hepatopancreas, intestines, gill, muscle, and hemocyte of shrimp were collected for viral load detection.

### Statistical Analysis

Data are expressed as mean ± standard deviation (SD). Statistical analyses were performed using SPSS software (version 18.0), with one-way ANOVA using Duncan's test to evaluate whether the means were significantly different (*P* < 0.05). Differences between groups were analyzed by using the Mantel–Cox (log-rank χ^2^ test) method with the GraphPad Prism software.

The formula for calculating the LD_50_ of the virus is:

$$\begin{array}{l} lgLD50\;=\;\frac{1}{2}\left(x_{i}\;+\;x_{i\;+\;1}\right)\left(\rho_{i\;+\;1}-\rho_{i}\right) \end{array}$$

where *x*_*i*_ is the logarithm of the dose or concentration and ρ_*i*_ is the mortality rate.

The 95% confidence interval for $LD50\;=\;lg^{-1}\;\left(lg\;Lc50\;\pm \;1.96\times S_{m}\right)$, where *S*_m_ is the standard error (32).

## Result

### LD_50_ of DIV1 for *L. vannamei*

**Figure 1:**
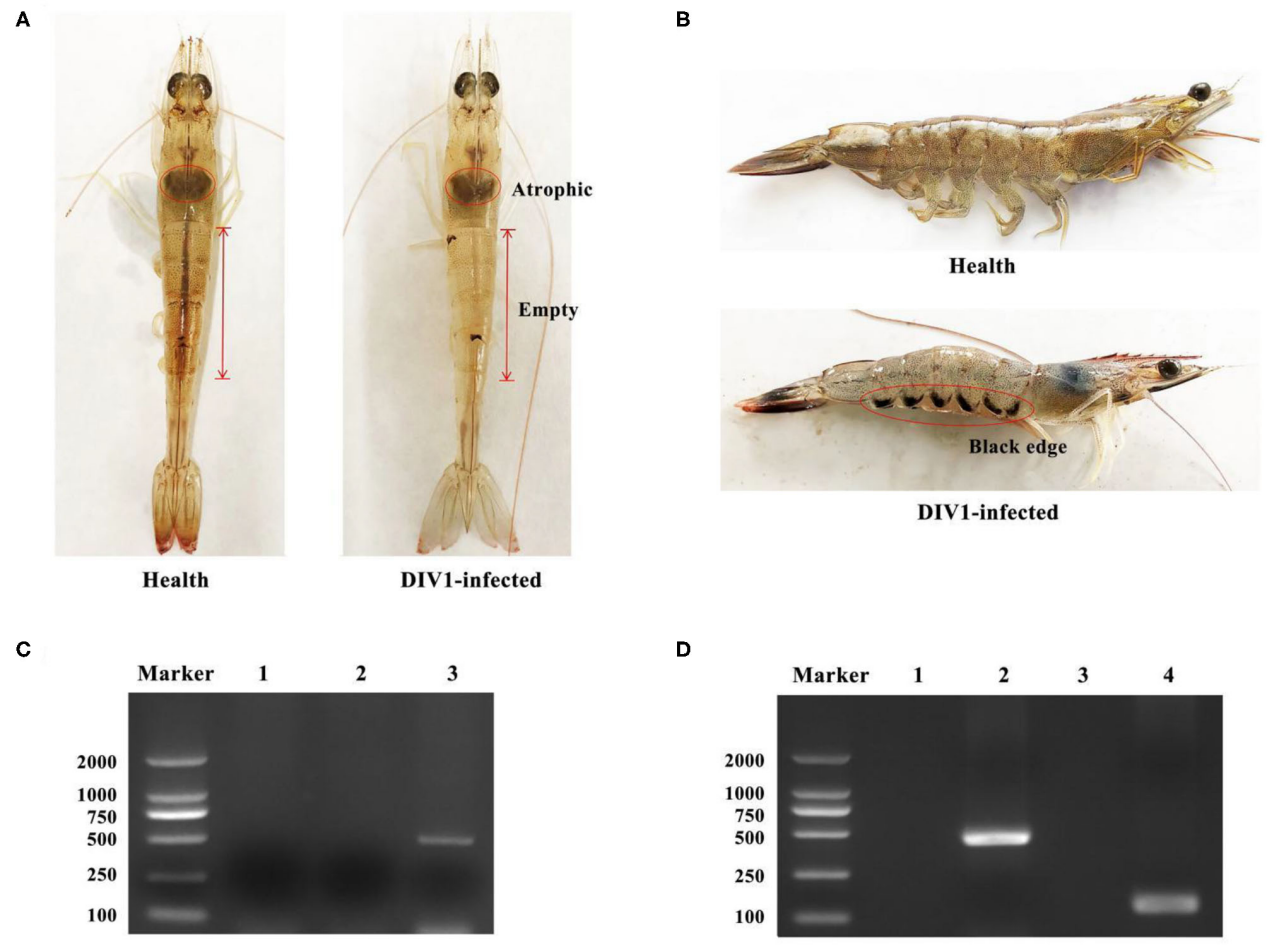
Clinical symptoms and virus detection of *Litopenaeus vannamei*. **(A,B)** Clinical symptoms of DIV1-infected *L. vannamei*. **(C)** Virus detection of infected *L. vannamei* used for the DIV1 inoculation in LD_50_ test. Marker: DL2000 molecular mass marker; lane 1: PCR amplified products of WSSV detection; lane 2: PCR-amplified products of IHHNV detection; lane 3: PCR amplified products of DIV1detection. **(D)** DIV1 detection of *L. vannamei* in LD_50_ test using nested PCR method. Marker: DL2000 molecular mass marker; lanes 1 and 3: PCR amplified products of DIV1 detection in healthy *L. vannamei*; lanes 2 and 4: PCR-amplified products of DIV1 detection in dead *L. vannamei*.

**Figure 2:**
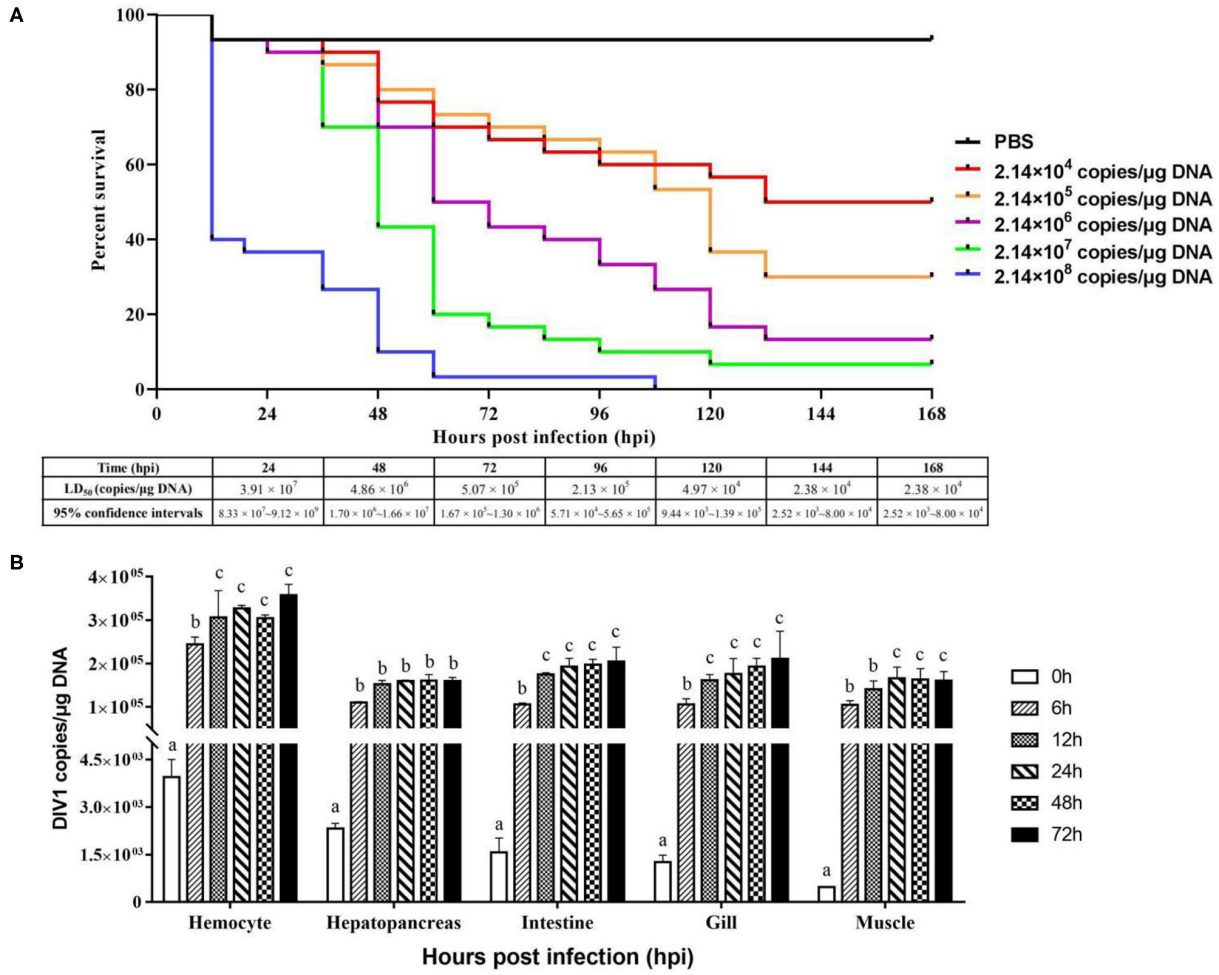
Cumulative survival rates of *Litopenaeus vannamei* injected by DIV1 **(A)** and genome copies of DIV1 in infected *L. vannamei* **(B)**. **(A)** Six groups of healthy *L. vannamei* were intramuscularly injected at the third abdominal segment with 50 μl of DIV1 supernatants at five concentrations and phosphate-buffered saline as a control. **(B)** The DIV1 copies were investigated in the hemocyte, hepatopancreas, intestine, gill, and muscle of *L. vannamei* infected by DIV1 at the concentration of LD50 after 48 h of infection. Dissimilar letters show a significant difference (*p* < 0.05).

*L. vannamei* had obvious symptoms after being infected with DIV1, including empty stomach and intestine in all diseased shrimp, atrophy, and lightening of hepatopancreas, and soft shell in partially infected shrimp (Figure 1A). Part of the dead shrimps because of DIV1 infection showed symptoms of black edge of the abdominal shell (Figure 1B). As shown in Figures 1C,D, only DIV1 was found in the infected *L. vannamei* used for the DIV1 inoculation and the dead *L. vannamei* in the LD_50_ test. Results on the survival rate of *L. vannamei* after exposure to different DIV1 concentrations and the copies of DIV1 in the *L. vannamei* at different DIV1 injections are shown in Figure 2. The shrimp mortality rate increased as the DIV1 concentration increased. Probit analysis showed that the LD_50_ values for DIV1 determined are 3.91 × 10^7^, 4.86 × 10^6^, 5.07 × 10^5^, 2.13 × 10^5^, 2.38 × 10^4^, and 2.38 × 10^4^ copies/μg DNA for 24, 48, 72, 96, 120, 144, and 168 h after injection, respectively (Figure 2A). The copies of DIV1 in all the five detected tissues of infected *L. vannamei* were significantly increased at all the detected timepoints after injection (Figure 2B).

### *De novo* Assembly and Annotation of Unigenes

**Figure 3:**
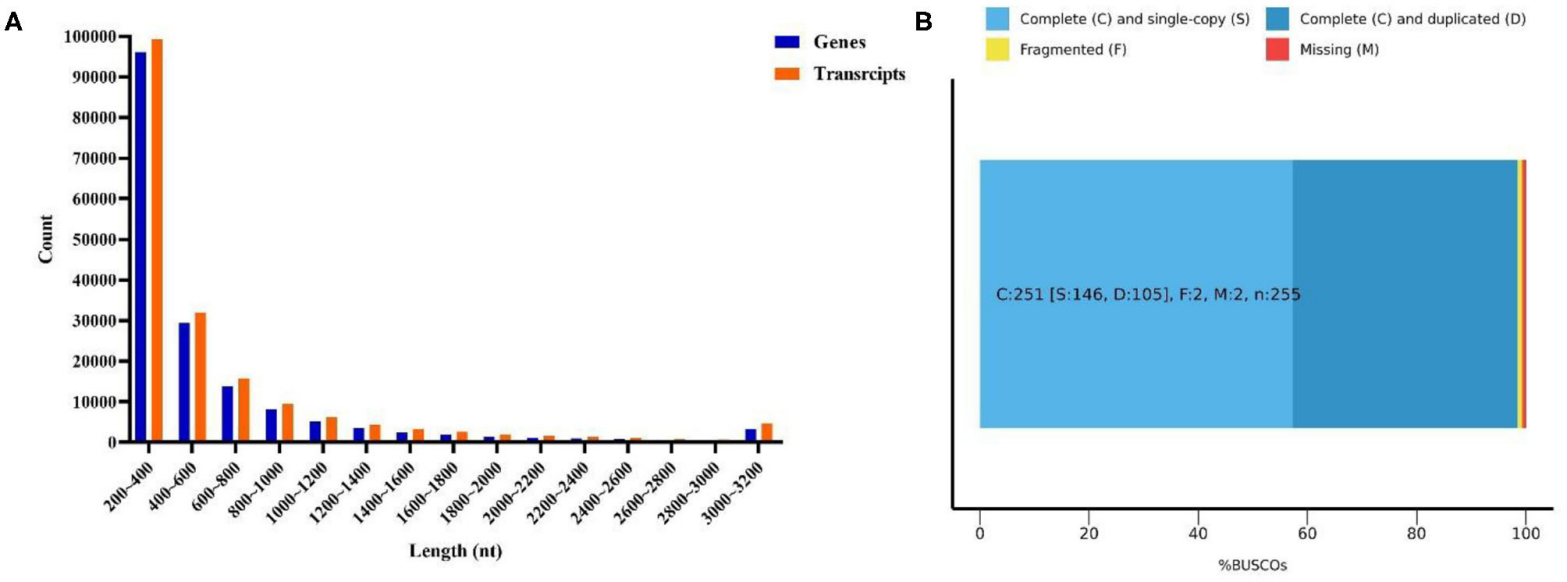
Transcriptome sequence length distribution **(A)** and assembly quality analysis **(B)**.

Six cDNA libraries from *L. vannamei* were sequenced on the Illumina HiSeqTM platform. As Table S2 shows, a total of 328,670,602 raw reads were generated, and 328,555,316 clean reads were left after removing the adapters filtering the low-quality sequences. Therefore, 163,056,696 clean reads were generated from 163,103,692 raw reads in the DIV1-infected group, and 165,498,620 clean reads were generated from 165,566,910 raw reads in the control group. The whole *de novo* assembly reads, from six libraries, yielded a total length of 101,529,805 bp, with 168,854 unigenes and an N50 length of 807 bp. The clean read data were deposited to the NCBI Sequence Read Archive (SRA, http://www.ncbi.nlm.nih.gov/Traces/sra) with the accession number SRP252506. A detailed summary of the sequencing and the assembly results is shown in Table 1. The sequence length (nt) ranges from 200 to ≥3,000 nt, with the distribution shown in Figure 3A. The most abundant unigenes were clustered in a group with 200–400 nt in length. Based on BUSCO, we compared the transcriptome with 1,066 conserved arthropod genes. A total of 98.4% of the transcriptome (251 genes) were encoded as complete proteins. Among these genes, 57.3% (146 genes) were complete and single-copy BUSCOs, 41.1% (105 genes) were complete and duplicated BUSCOs, 0.8% (two genes) were fragmented BUSCOs, and 0.8% (two genes) were missing BUSCOs (Figure 3B).

**Table 1:** Summary of *de novo* assembly of *Litopenaeus vannamei* hemocyte transcriptome.

**Type**	**Total number (*n*)**	**Total length (nt)**	**Mean length (nt)**	**N50 (bp)**	**GC (%)**
Genes	168,854	101,529,805	601	807	44.5415
Transcripts	185,058	123,045,518	664	975	44.7676

### Functional Annotation and Classification of Unigenes

All unigenes were annotated using BLASTx with the NCBI nonredundant (Nr), KEGG, SWISS-PROT, and KOG protein database. Annotation information was retrieved from proteins with the highest sequence similarity. In this study, 62,270 (36.88%) unigenes were annotated. Among them, a total of 48,135, 28,835, 53,506, and 43,824 unigenes were annotated in the Nr, KEGG, SWISS-PROT, and KOG database, respectively (Table 2). The Blast hits a total of 940 species, the top five of which were *Branchiostoma belcheri* (5,329, 11.07%), *Hyalella azteca* (4,687, 9.74%), *Saccoglossus kowalevskii* (2,350, 4.88%), *Lingula anatine* (1,954, 4.06%), and *Limulus polyphemus* (1,266, 2.62%).

**Table 2:** Annotation of unigenes from transcriptome.

**Values**	**Total**	**Nr**	**KEGG**	**SWISS-PROT**	**KOG**	**Annotated**	**Without annotation**
Number	168,854	48,135	28,835	53,506	43,824	62,270	106,584
Percentage	100%	28.51%	17.08%	31.69%	25.95%	36.88%	63.12%

**Figure 4:**
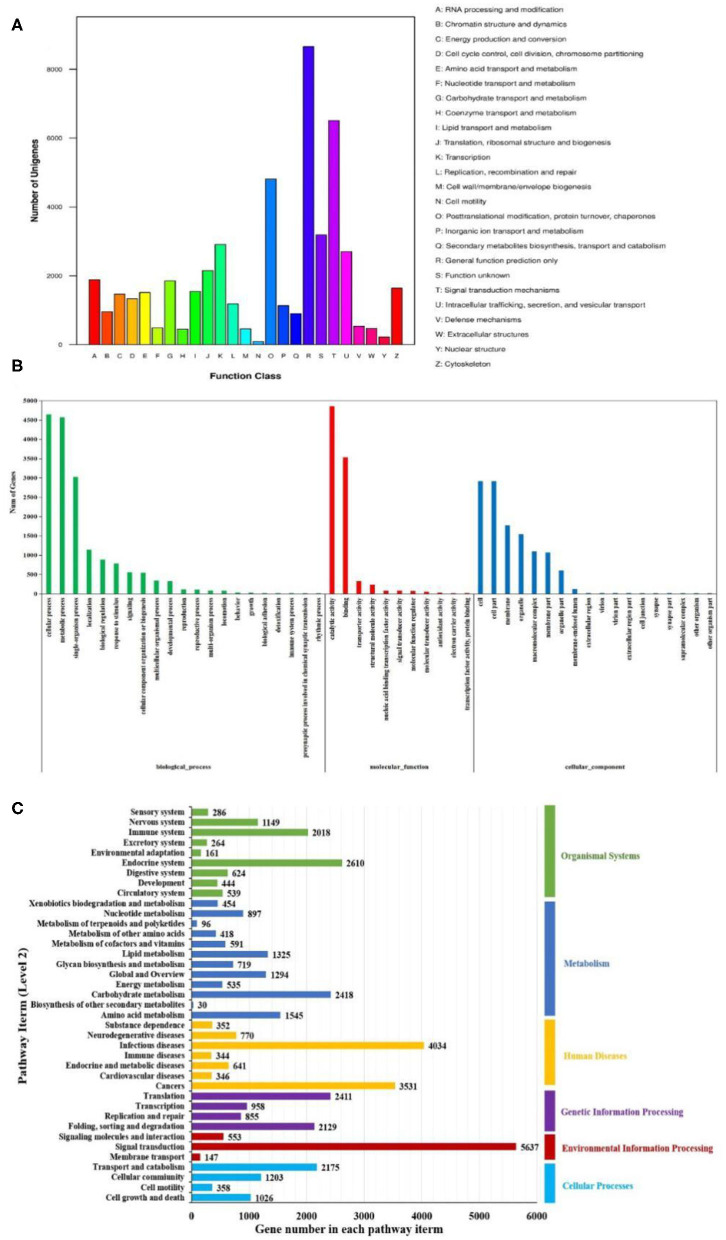
Functional enrichment of unigenes from *Litopenaeus vannamei*. **(A)** KOG classification of unigenes. Each bar represents the number of unigenes classified into each of the 26 KOG functional categories. **(B)** Gene Ontology (GO) classification of unigenes. Three major GO categories were enriched: biological process, cellular component, and molecular function. **(C)** Kyoto Encyclopedia of Genes and Genomes (KEGG) classification of unigenes. The unigenes were assigned to six special KEGG pathways, including organismal systems, metabolism, human diseases, genetic information processing, environmental information processing, and cellular processes.

The KOG analysis showed that 49,048 unigenes were classified into 25 functional categories (Figure 4A). The largest three groups were “general function prediction only” (8,658, 17.65%), “signal transduction mechanisms” (6,511, 13.27%), and “posttranslational modification, protein turnover, chaperones” (4,809, 9.81%). The smallest cluster was “cell motility,” which only contained 91 unigenes. By GO analysis, 17,321, 8,281, and 12,146 unigenes were classified into biological process, molecular function, and cellular component by Blast2GO suite, respectively (Figure 4B). Within the biological process category, “cellular process” (4,644 unigenes) and “metabolic process” (4,566 unigenes) were the dominant groups. Within the cellular component category, “cell” (2,909 unigenes) and “cell part” (2,909 unigenes) were the most abundant groups. Within the molecular function category, “catalytic activity” (4,859 unigenes) and “binding” (3,534 unigenes) were the dominant groups. Using KEGG, a total of 15,902 unigenes were mapped to six specific pathways, including cellular processes, environmental information processing, genetic information processing, metabolism, human diseases, and organism system (Figure 4C). These annotated unigenes were further divided into 39 level 2 subcategory pathways. The largest subcategory group, signal transduction, had 5,637 annotated genes, followed by infection diseases (4,034), cancers (3,531), the endocrine system (2,610), carbohydrate metabolism (2,418), and translation (2,412). Apart from these, 302 level 3 KEGG subcategories were annotated and are listed in Table S3.

### Classification and Analysis of DEGs

**Figure 5:**
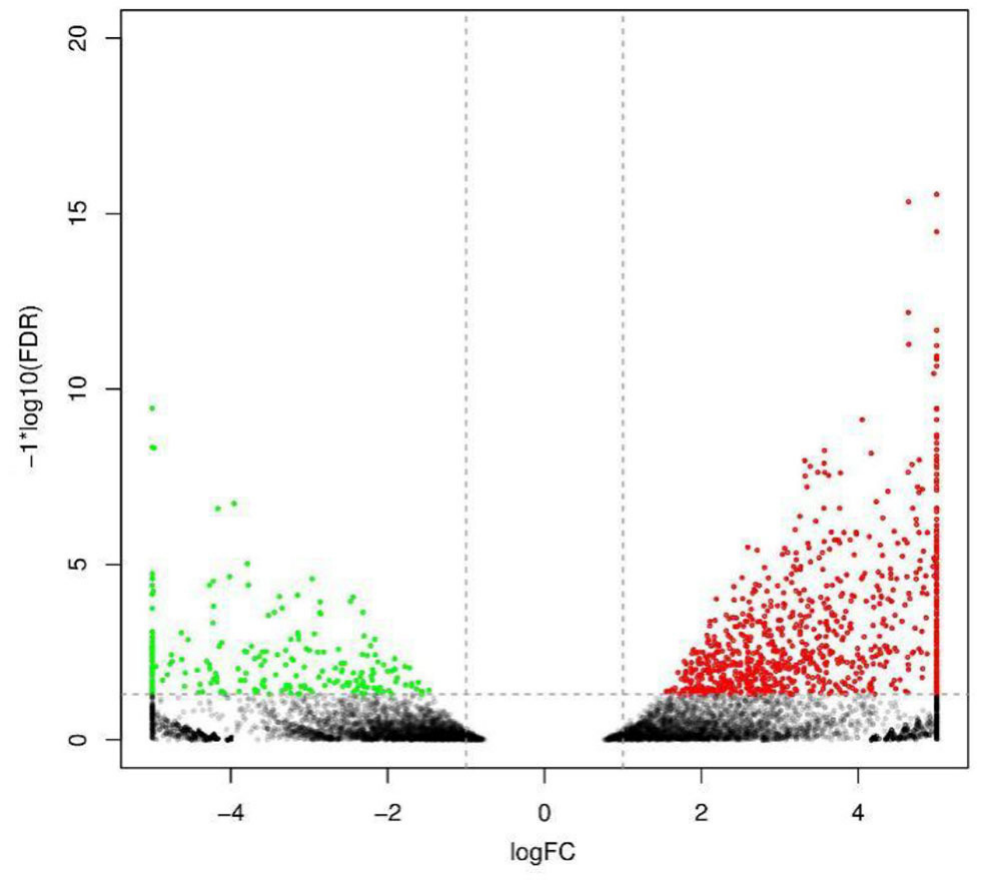
Volcano diagram of differentially expressed genes (DEGs) in *Litopenaeus vannamei* with and without DIV1 infection. The x-axis indicates the fold change, and the y-axis indicates the statistical significance of the differences. Red dots represent the significantly up-regulated DEGs, while green dots represent the significantly down-regulated DEGs (FDR < 0.05 and |log2 ratio| ≥ 1). The gray dots represent the DEGs which are not significantly different.

To analyze and characterize the DEGs in *L. vannamei* following DIV1 infection, a cutoff false discovery rate (FDR) was set at < 0.05 and a |log_2_ ratio| ≥1 was employed as threshold. Based on this, 1,112 genes were observed to be dysregulated in DIV1-infected group compared to the control, including 889 up-regulated genes and 223 down-regulated genes. These DEGs were visualized by volcano plot in Figure 5.

**Figure 6:**
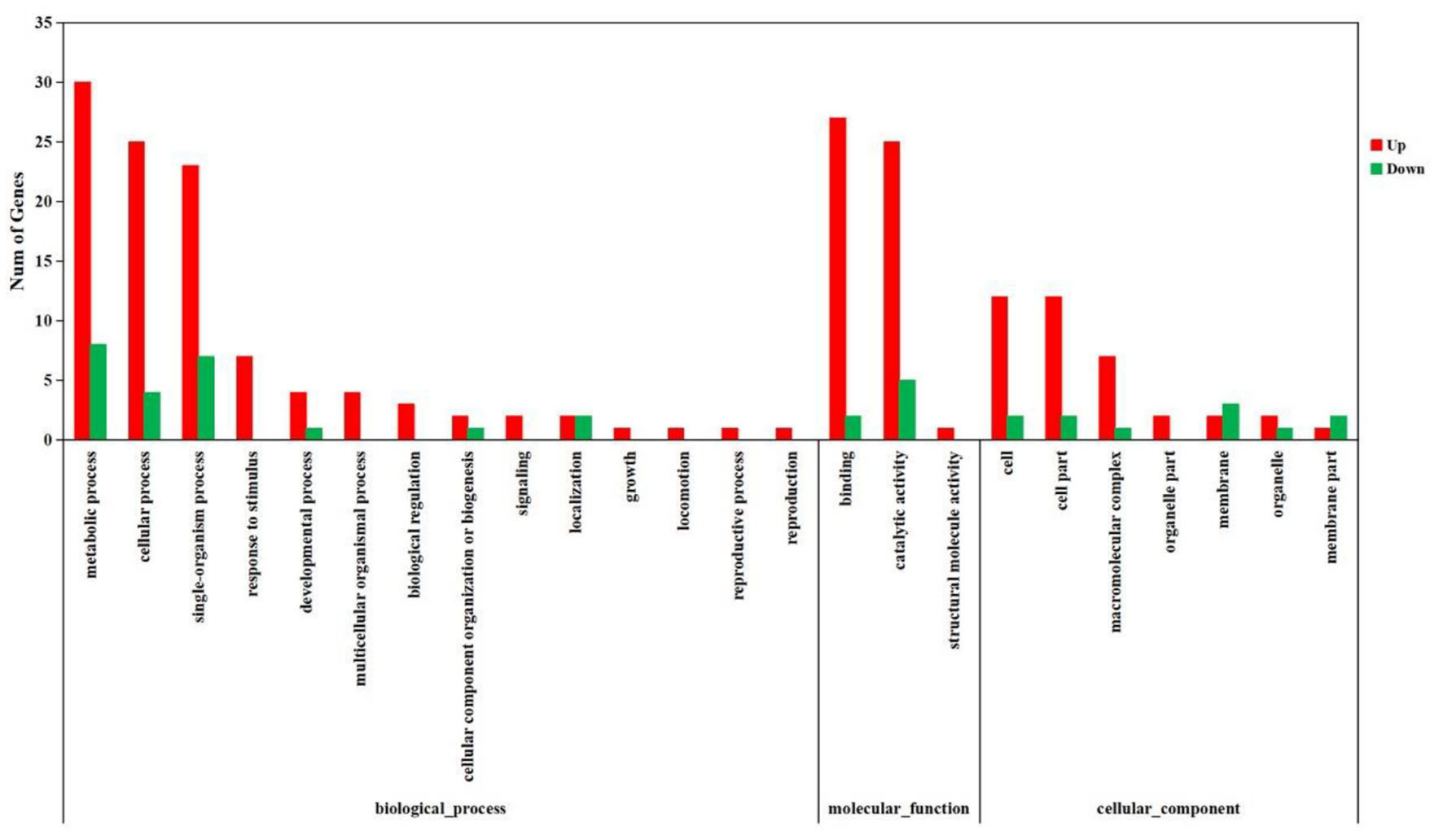
Analysis of GO term functional enrichment of differentially expressed genes between DIV1-infected and control groups. The x-axis indicates the Gene Ontology processes, and the y-axis indicates the number of unigenes in a process.

**Figure 7:**
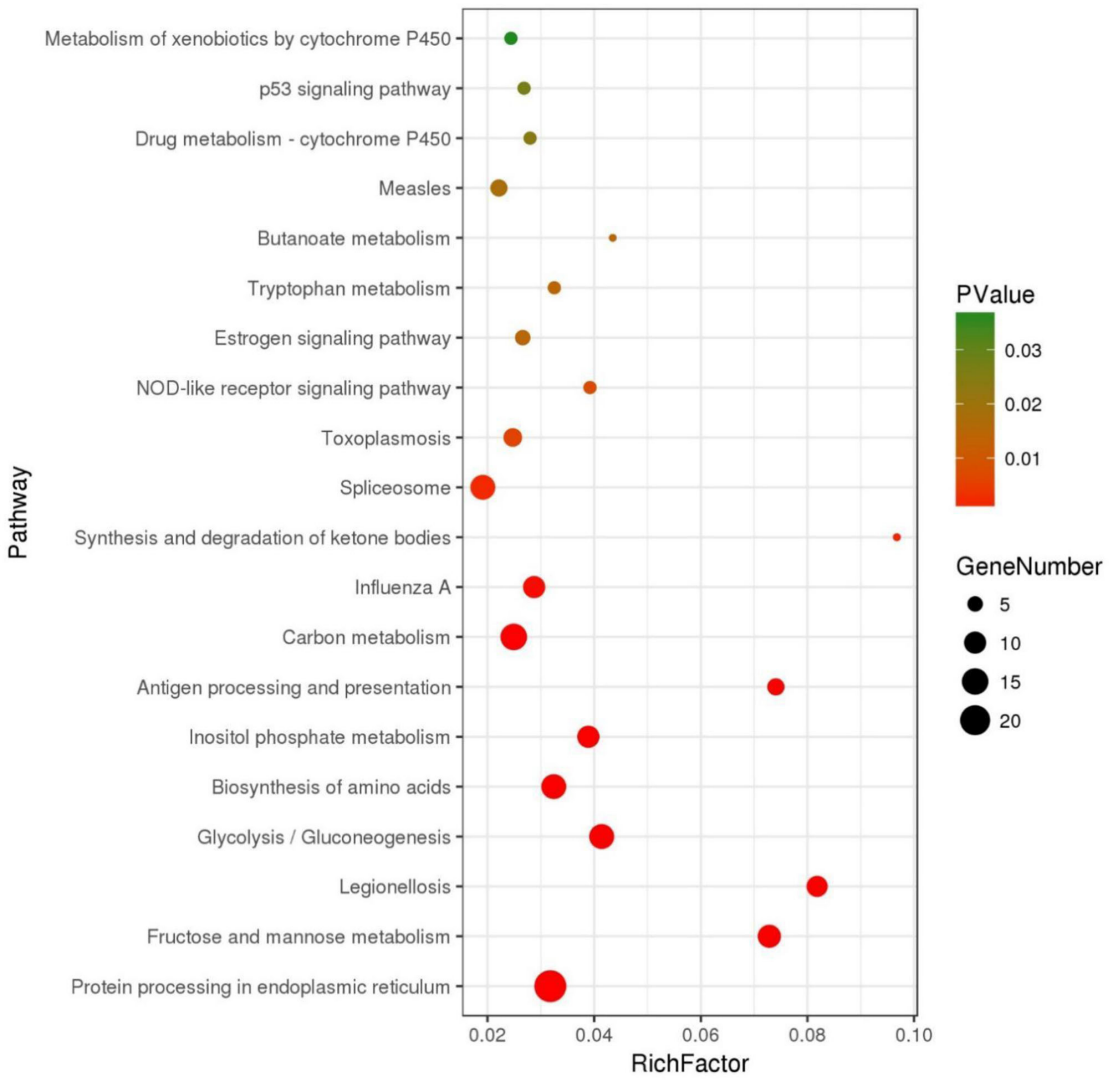
Top 20 of pathway enrichment. The x-axis indicates the ratio of the number of genes in the pathway of the DEGs and all genes. The y-axis indicates the pathway.

The DEGs were further annotated with GO and KEGG databases. In the GO enrichment analysis, the 197 up-regulated and the 41 down-regulated genes expressed in the DIV1-infected group were enriched in several categories: biological process (106 up-regulated and 23 down-regulated), molecular function (38 up-regulated and 11 down-regulated), and cellular component (53 up-regulated and seven down-regulated) (Figure 6). For the KEGG pathway enrichment analysis, 121 DEGs were annotated into 108 pathways. Among them, metabolism was a crucial pathway. The category that contained the higher number of DEGs was “protein processing in endoplasmic reticulum.” The top 20 KEGG enrichment pathways influenced by DIV1 infection are shown in Figure 7. KEGG analysis showed that 28 DEGs were presented in seven immune system pathways, including NOD-like receptor signaling pathway (four), MAPK signaling pathway (seven), Wnt signaling pathway (two), Toll-like receptor signaling pathway (two), phagosome (seven), RIG-I-like receptor signaling pathway (two), and p53 signaling pathway (four) (Table 3). In these pathways, *TPI* genes received particular attention for their participation in several distinct pathways, such as fructose and mannose metabolism, glycolysis/gluconeogenesis, biosynthesis of amino acids, inositol phosphate metabolism, and carbon metabolism (Table 4).

**Table 3:** Differentially expressed genes associated with immune responses during DIV1 infection.

**Category or gene ID**	**Gene description**	**Species**	**FC^a^**
**NOD-like receptor signaling pathway**			
Unigene024064_All	Endoplasmin	*Bemisia tabaci*	2.64
Unigene056132_All	Caspase-2	*Cerapachys biroi*	1.96
Unigene067577_All	NACHT, LRR, and PYD domain-containing protein 3-like	*Branchiostoma belcheri*	4.64
Unigene072846_All	Protein NLRC5-like	*Acropora digitifera*	3.60
**MAPK signaling pathway**			
Unigene011119_All	Cytosolic heat shock protein 70, partial	*Mytilus galloprovincialis*	6.08
Unigene039540_All	Heat shock protein 70 kDa, partial	*Bythograea thermydron*	2.83
Unigene045522_All	Heat shock cognate protein 70, partial	*Latrodectus hesperus*	3.50
Unigene055746_All	70-kDa heat shock protein C, partial	*Euphausia superba*	3.72
Unigene055749_All	High-molecular-weight heat shock protein	*Acanthamoeba castellanii str. Neff*	2.52
Unigene055750_All	Heat shock cognate protein 70	*Haliotis diversicolor*	2.53
Unigene123680_All	NPKL2	*Oryza sativa Japonica Group*	3.83
**Wnt signaling pathway**			
Unigene031735_All	Calcyclin-binding protein-like	*Parasteatoda tepidariorum*	5.67
Unigene031736_All	SGS domain-containing protein	*Toxoplasma gondii*	7.07
**Toll-like receptor signaling pathway**			
Unigene056132_All	Caspase-2	*Cerapachys biroi*	1.96
Unigene137202_All	Interleukin-1 receptor-associated kinase 4-like	*Parasteatoda tepidariorum*	3.59
**Phagosome**			
Unigene001107_All	Calnexin-like protein	*Littorina littorea*	2.32
Unigene025407_All	Calreticulin	*Dictyostelium lacteum*	1.98
Unigene047081_All	C-type lectin	*Litopenaeus vannamei*	6.68
Unigene061835_All	Thrombospondin II	*Penaeus monodon*	7.68
Unigene068957_All	Cathepsin L	*Penaeus monodon*	2.93
Unigene117499_All	Ervatamin-B	*Oryza sativa japonica* group	−4.25
Unigene155244_All	Cathepsin L-like cysteine proteinase	*Longidorus elongatus*	−3.34
**RIG-I-like receptor signaling pathway**			
Unigene041866_All	ATP-dependent RNA helicase DDX3X-like protein	*Rhinopithecus roxellana Saccoglossus kowalevskii*	5.54
Unigene056132_All	Caspase-2	*Cerapachys biroi*	1.96
**p53 signaling pathway**			
Unigene018326_All	Cytochrome c-like isoform X1	*Galendromus occidentalis*	3.26
Unigene027278_All	Ribonucleoside-diphosphate reductase subunit M2 B-like	*Limulus polyphemus*	−4.54
Unigene056132_All	Caspase-2	*Cerapachys biroi*	1.96
Unigene064926_All	Cytochrome c	*Litopenaeus vannamei*	2.73

a *Fold changes (log_2_ ratio) in expression*.

**Table 4:** Triosephosphate isomerase genes and the pathways and the genes related to them in differentially expressed genes.

**Category or gene ID**	**Gene description**	**Species**	**FC^a^**
**Fructose and mannose metabolism**			
Unigene015918	CLUMA_CG013551, isoform A	*Clunio marinus*	2.79
Unigene068542	Triosephosphate isomerase	*Penaeus monodon*	4.31
Unigene068543	Triosephosphate isomerase	*Palaemon carinicauda*	4.83
Unigene046663	Fructose-bisphosphate aldolase	*Dictyostelium lacteum*	−5.41
Unigene051281	Triosephosphate isomerase	*Penaeus monodon*	5.22
Unigene064463	Triosephosphate isomerase	*Litopenaeus vannamei*	5.17
Unigene064464	Triosephosphate isomerase	*Penaeus monodon*	5.25
Unigene064465	Triosephosphate isomerase	*Penaeus monodon*	5.21
Unigene068538	Triosephosphate isomerase	*Penaeus monodon*	3.90
Unigene068539	Triosephosphate isomerase	*Penaeus monodon*	4.42
Unigene068541	Triosephosphate isomerase	*Penaeus monodon*	4.29
**Glycolysis/gluconeogenesis**			
Unigene038694	Multiple inositol polyphosphate phosphatase	*Daphnia magna*	−2.59
Unigene046663	Fructose-bisphosphate aldolase	*Dictyostelium lacteum*	−5.41
Unigene051281	Triosephosphate isomerase	*Penaeus monodon*	5.22
Unigene062010	Phosphoenolpyruvate carboxykinase	*Litopenaeus vannamei*	3.92
Unigene064463	Triosephosphate isomerase	*Litopenaeus vannamei*	5.17
Unigene064464	Triosephosphate isomerase	*Penaeus monodon*	5.25
Unigene064465	Triosephosphate isomerase	*Penaeus monodon*	5.21
Unigene068538	Triosephosphate isomerase	*Penaeus monodon*	3.90
Unigene068539	Triosephosphate isomerase	*Penaeus monodon*	4.42
Unigene068541	Triosephosphate isomerase	*Penaeus monodon*	4.29
Unigene068542	Triosephosphate isomerase	*Penaeus monodon*	4.31
Unigene068543	Triosephosphate isomerase	*Palaemon carinicauda*	4.83
Unigene074167	Acetyl-coenzyme A synthetase 2-like, mitochondrial	*Crassostrea gigas*	1.79
**Biosynthesis of amino acids**			
Unigene018569	Kynurenine aminotransferase 4	*Dictyostelium discoideum AX4*	2.95
Unigene046663	Fructose-bisphosphate aldolase	*Dictyostelium lacteum*	−5.41
Unigene051281	Triosephosphate isomerase	*Penaeus monodon*	5.22
Unigene059719	S-adenosylmethionine synthetase	*Polysphondylium pallidum PN500*	2.09
Unigene064463	Triosephosphate isomerase	*Litopenaeus vannamei*	5.17
Unigene064464	Triosephosphate isomerase	*Penaeus monodon*	5.25
Unigene064465	Triosephosphate isomerase	*Penaeus monodon*	5.21
Unigene068538	Triosephosphate isomerase	*Penaeus monodon*	3.90
Unigene068539	Triosephosphate isomerase	*Penaeus monodon*	4.42
Unigene068541	Triosephosphate isomerase	*Penaeus monodon*	4.29
Unigene068542	Triosephosphate isomerase	*Penaeus monodon*	4.31
Unigene068543	Triosephosphate isomerase	*Palaemon carinicauda*	4.83
Unigene083661	Phosphoserine aminotransferase, chloroplastic	*Sphaeroforma arctica JP610*	2.81
**Inositol phosphate metabolism**			
Unigene038694	Multiple inositol polyphosphate phosphatase	*Daphnia magna*	−2.59
Unigene051281	Triosephosphate isomerase	*triosephosphate isomerase*	5.22
Unigene064463	Triosephosphate isomerase	*Litopenaeus vannamei*	5.17
Unigene064464	Triosephosphate isomerase	*Penaeus monodon*	5.25
Unigene064465	Triosephosphate isomerase	*Penaeus monodon*	5.21
Unigene068538	Triosephosphate isomerase	*Penaeus monodon*	3.90
Unigene068539	Triosephosphate isomerase	*Penaeus monodon*	4.42
Unigene068541	Triosephosphate isomerase	*Penaeus monodon*	4.29
Unigene068542	Triosephosphate isomerase	*Penaeus monodon*	4.31
Unigene068543	Triosephosphate isomerase	*Palaemon carinicauda*	4.83
**Carbon metabolism**			
Unigene018569	Aspartate aminotransferase	*Dictyostelium discoideum AX4*	2.95
Unigene046663	Fructose-bisphosphate aldolase	*Dictyostelium lacteum*	−5.41
Unigene051281	Triosephosphate isomerase	*Penaeus monodon*	5.22
Unigene061564	Putative uncharacterized protein DDB_G0277255	*Hyalella azteca*	2.35
Unigene064463	Triosephosphate isomerase	*Litopenaeus vannamei*	5.17
Unigene064464	Triosephosphate isomerase	*Penaeus monodon*	5.25
Unigene064465	Triosephosphate isomerase	*Penaeus monodon*	5.21
Unigene068538	Triosephosphate isomerase	*Penaeus monodon*	3.90
Unigene068539	Triosephosphate isomerase	*Penaeus monodon*	4.42
Unigene068541	Triosephosphate isomerase	*Penaeus monodon*	4.29
Unigene068542	Triosephosphate isomerase	*Penaeus monodon*	4.31
Unigene068543	Triosephosphate isomerase	*Palaemon carinicauda*	4.83
Unigene074167	Acetyl-coenzyme A synthetase 2-like, mitochondrial	*Crassostrea gigas*	1.79
Unigene083661	Phosphoserine aminotransferase, chloroplastic	*Sphaeroforma arctica JP610*	2.81
Unigene150662	Malate dehydrogenase	*malate dehydrogenase*	3.23

a *Fold changes (log_2_ ratio) in expression*.

### Validation of RNA-Seq Results by qRT-PCR

**Figure 8:**
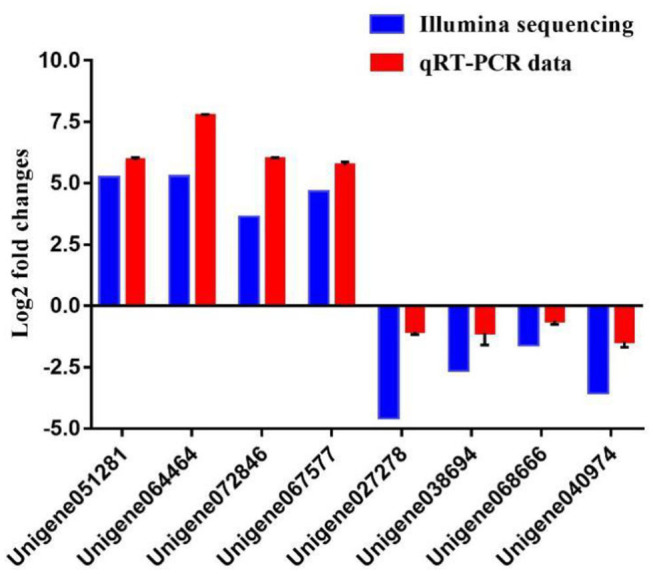
Comparison of the expression profiles of six selected genes as determined by Illumina sequencing and qRT-PCR.

To further evaluate our DEG library, eight unigenes were randomly selected, including four up-regulated and four down-regulated DEGs for qPCR analysis. The amplification efficiency (*E*) of all unigenes and EF1α ranged from 95.2 to 98.2% (Table S1). As shown in Figure 8, the qPCR results showed significant, identical expression tendencies as the high-throughput sequencing data. However, some quantitative differences in the expression level were seen for Unigene064464, Unigene072846, Unigene027278, and Unigene040974. The qPCR analysis results, therefore, confirmed the expressions of DEGs which were detected in the high-throughput sequencing analysis.

### Functional Analysis of *LvTPI* in *L. vannamei* During DIV1 Infection

#### Silencing of *LvTPI-likes* Led to *L. vannamei* Death

**Figure 9:**
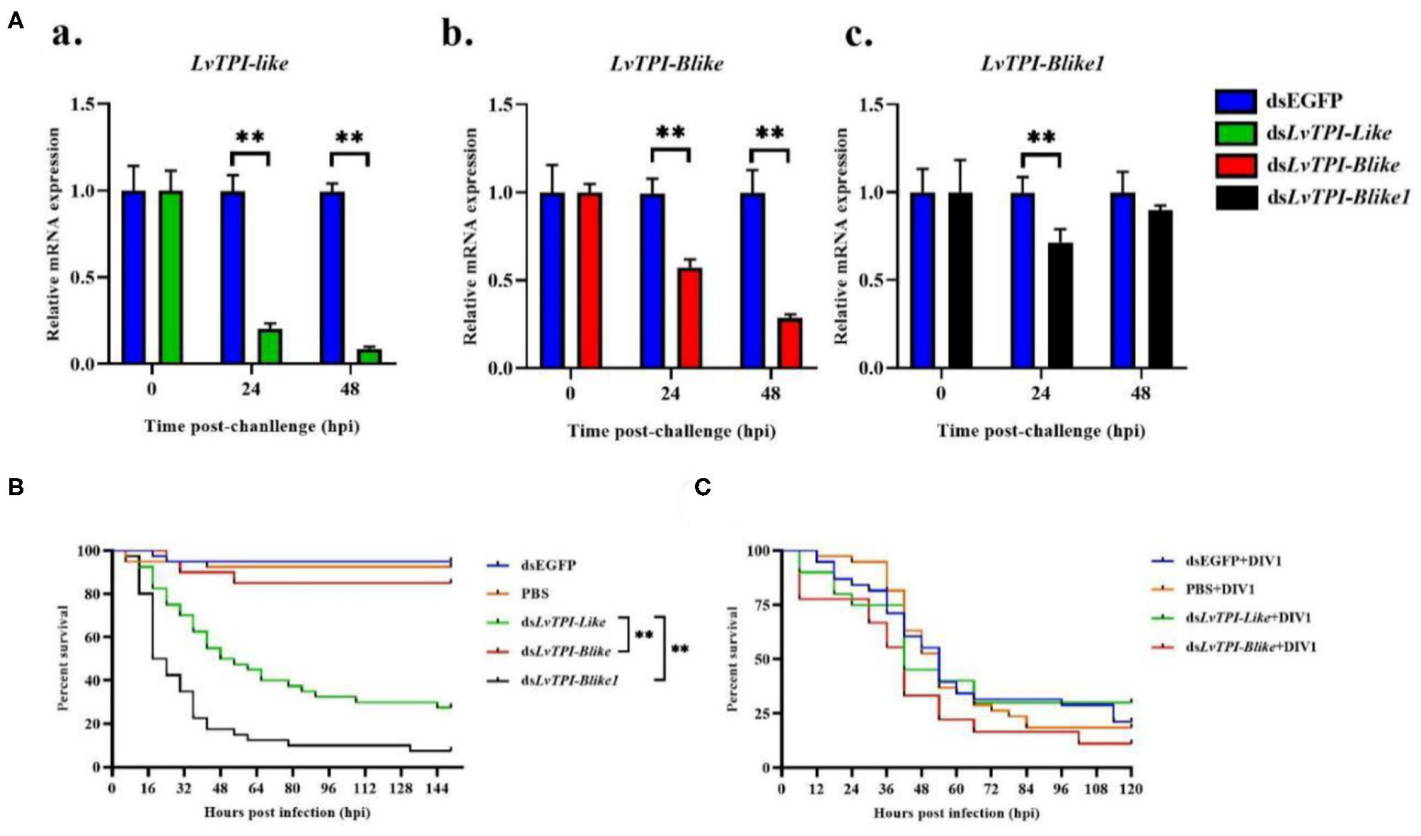
Function of *LvTPI-likes* during DIV1 infection. **(A)** qPCR analysis of the silencing efficiencies of *LvTPI-likes*. (a) *LvTPI-like*, (b) *LvTPI-Blike*, and (c) *LvTPI-Blike1*. EF1α was used as the internal control. **(B)** Cumulative survival rates of *Litopenaeus vannamei* injected by *LvTPI-likes* dsRNA. **(C)** Cumulative survival rates of *LvTPI-likes*-RNAi *L. vannamei* during DIV1 infection. Error bars represent ± SD of three replicates. Data were analyzed with the GraphPad Prism software using the log-rank (Mantel–Cox) method. All data are given in terms of means ± standard error (SE). Asterisks indicate significant differences. *P < 0.05 and **P < 0.01 (*n* = 3).

According to the transcriptome information in this study and the genome information from NCBI, three full-length TPI types were obtained and named as *LvTPI-like* (accession no. MT123334), *LvTPI-Blike* (accession no. MT107901), and *LvTPI-Blike1* (accession no. MN996302). The silencing efficiency was checked using qPCR. At 24 and 48 h post-dsRNA injection, the mRNA level of *LvTPI-likes* was remarkably downregulated in dsRNA-*LvTPI-likes*-treated shrimp (*p* < 0.05), whereas there was no suppressive effect on *LvTPI-likes* in the dsRNA-EGFP-treated group (Figure 9A). The *L. vannamei* started to die after dsRNA-*LvTPI-like* and dsRNA-*LvTPI-Blike1* injection, with a cumulative mortality of 50 and 82.5% at 48 hpi, respectively. The final mortality rates at 144 hpi were 72.5 and 92.5% for the dsRNA-*LvTPI-like* and the dsRNA-*LvTPI-Blike1* groups, respectively. However, there was no effect on the survival rate of *L. vannamei* by dsRNA-*LvTPI-Blike* injection (Figure 9B).

#### *LvTPI-like* and *LvTPI-Blike* Suppression Did Not Affect the Survival Rates of *L. vannamei* but Reduced DIV1 Replication

**Figure 10:**
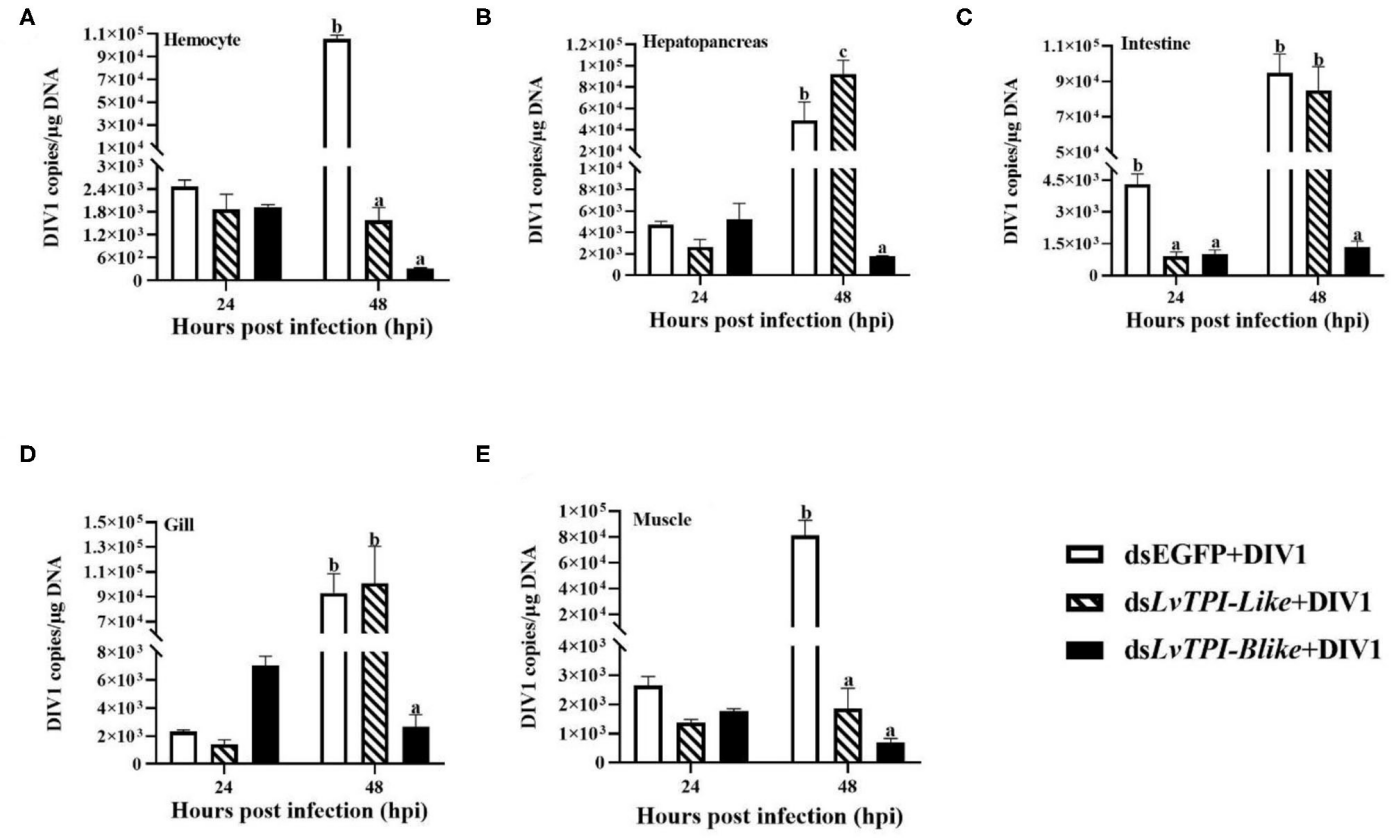
Detection of DIV1 copy numbers in hemocyte **(A)**, hepatopancreas **(B)**, intestine **(C)**, gill **(D)**, and muscle **(E)** of *Litopenaeus vannamei* following treatment with ds*LvTPI-like* and *dsLvTPI-Blike*. Data are shown as mean ± SD of three animals. Dissimilar letters show a significant difference (*p* < 0.05).

Due to the mass death of *L. vannamei* when *LvTPI-Blike1* was silenced, the function of *LvTPI-like* and *LvTPI-Blike* in DIV1-infected *L. vannamei* was investigated. The shrimp were challenged with DIV1 at 48 h post-dsRNA injection in the following experiments. As shown in Figure 9C, the cumulative survival rate in the dsRNA-*LvTPI-Blike* group was lower than those in the dsRNA-EGFP group. However, there was no significant difference between the cumulative survival rate of the dsRNA-*LvTPI-like* and dsRNA-*LvTPI-Blike* groups compared with the dsRNA-EGFP group during DIV1 infection. The final survival rates were 30.0, 11.1, and 28.95% for dsRNA-*LvTPI-like*, dsRNA-*LvTPI-Blike*, and dsRNA-EGFP groups, respectively. However, both dsRNA-*LvTPI-like* and dsRNA-*LvTPI-Blike* suppression reduced DIV1 replication. The virus copies in five tissues—hemocyte, hepatopancreas, intestine, gill, and muscle—were measured in shrimp at 24 and 48 h post-DIV1 infection in each double-stranded RNA silencing group. As shown in Figure 10, the DIV1 copy numbers for both the dsRNA-*LvTPI-like* and the dsRNA-*LvTPI-Blike* groups were not significantly different from that for the dsRNA-EGFP group at 24 hpi. At 48 hpi, the viral loads in the hemocyte, hepatopancreas, intestine, gill, and muscle of the ds*LvTPI-Blike* + DIV1 group was 3.10 × 10^2^, 1.75 × 10^3^, 1.34 × 10^3^, 2.64 × 10^3^, and 6.95 × 10^2^ copies/μg DNA, respectively. The viral loads in all the detected tissues of the ds*LvTPI-Blike* + DIV1 group were significantly lower than those of the dsRNA-EGFP + DIV1 control group (*p* < 0.05). In the ds*LvTPI-like* + DIV1 group, the number of copies of DIV1 at 48 hpi decreased in the hemocyte and muscle but increased in the hepatopancreas, intestine, and gill, with a significantly different level only in the hepatopancreas.

## Discussion

DIV1 is a new disease prevalent in shrimp cultures in China. DIV1 mainly affects the hematopoietic tissue and the hemocytes of shrimp (6). The emergence of DIV1 poses a new biological risk to the shrimp farming industry (33). However, there are no reports on the harmful effects of DIV1 on *L. vannamei* until now. In this study, the toxicity of DIV1 for *L. vannamei* was measured at different time points, and a comparative transcriptome analysis of *L. vannamei* challenged by DIV1 was conducted. The results showed that several metabolisms and immune function signaling pathways participated in the *L. vannamei* response to DIV1. In addition, *TPI* genes play an outstanding role.

As an important parameter of virulence, LD_50_ was often used to evaluate the effect of virus on shrimp. In crustaceans, the LD_50_ of several disease-causing viruses including WSSV, IHHNV, and TSV have been reported (34–36); however, the LD_50_ of DIV1 in shrimp has not been found. To our knowledge, this is the first report on the virulence of DIVI in crustaceans. The detection of DIV1 replication in LD_50_ test showed that the copies of DIV1 in the hemocyte, hepatopancreas, intestine, gill, and muscle of infected *L. vannamei* were significantly increased at all the detected timepoints after injection. A consistent conclusion was confirmed by Qiu et al. A histological analysis of ultrathin sections imaged under transmission electron microscopy revealed that enveloped icosahedral virus-like particles were present in hemocytes localized to the hemal sinus, hepatopancreas, and muscle of *L. vannamei* infected by DIV1 (6). It can be inferred that the mortality of *L. vannamei* was the result of virus replication.

Transcriptome sequencing, a powerful tool in biological research, has been used to analyze the immune response to many shrimp pathogens (37). Xue et al. compared the transcriptome profiles in hemocytes of uninfected and WSSV-infected *L. vannamei* and found 1,179 immune-related unigenes (38). Zeng et al. performed transcriptome sequencing in the hepatopancreas of *L. vannamei* infected with TSV and found 1,311 differential genes, including a large number of immune-related genes (39). Hui et al. used transcriptome analysis to reveal a large number of immune-related genes in the intestine of *M. rosenbergii* infected with WSSV (40). By using transcriptome sequencing, Cao et al. also obtained an abundant number of immune-related genes, such as toll-like receptors, C-type lectins, and scavenger receptors, during WSSV infection of *M. rosenbergii* (41). In the present study, transcriptome analysis was conducted to identify genes and pathways in *L. vannamei* which may play a role during the infection of DIV1. Similarly, a large number of immune-related genes were found, participating in several immune-related pathways, including NOD-like receptor, MAPK, Wnt, Toll-like receptor, phagosome, RIG-I-like receptor, and p53 signaling pathways. The results indicated that immune response was necessary when the shrimps suffered the attacks of the virus. It is noteworthy that KEGG analysis showed that several metabolism-related pathways such as fructose and mannose metabolism, glycolysis/gluconeogenesis, biosynthesis of amino acids, inositol phosphate metabolism, and carbon metabolism are members of the top 20 KEGG enrichment pathways influenced by DIV1 infection. A previous study showed that the replication and the packaging of DIV1 not only requires nucleic acids and proteins but also phospholipids to form the inner limiting envelope (6). Thinking of the increased copies of DIV1 in the infected *L. vannamei*, it can be seen that infection with DIV1 results in a metabolic disorder of *L. vannamei*, which supported the general model of viral pathogenesis causing a systemic disruption to metabolic pathways as the host physiology is taken over to support viral replication (42). Consistent results have been reports in other two crustacean species, *C. quadricarinatus* and *F. merguiensis*. Yang et al. found that DIV1 infection induced changes in carbohydrate metabolism, lipid metabolism, and amino acid metabolism in *C. quadricarinatus* (13). Our previous study in *F. merguiensis* found that DIV1 affected not only some immune-related pathways but also some metabolic pathways such as amino sugar and nucleotide sugar metabolism, glycolysis/gluconeogenesis, and inositol phosphate metabolism (14). However, the key factors that play important roles during DIV1 infection in these species are still unclear.

Another notable result in our transcriptome analysis was that lots of DEGs annotated as TPI participated in several members of the top 20 KEGG enrichment pathways. *TPI* was known as an important factor which plays a role in both glycolysis and phospholipid biosynthesis in all organisms (43). *TPI* is an enzyme in the glycolytic pathway, which catalyzes the reversible interconversion of dihydroxyacetone phosphate (DHAP) and the triose phosphate glyceraldehyde 3-phosphate (GAP) (44–47). *TPI* is vital to an organism's response to external factors (48). Besides that, *TPI* could be used to develop vaccines against parasitic diseases in mammals (49, 50). In shrimp, the only study in *Exopalaemon carinicauda* showed that TPI facilitated the replication of WSSV (51). In this study, three types of TPI genes (namely, *LvTPI-like, LvTPI-Blike*, and *LvTPI-Blike1*) were obtained in *L. vannamei*, and their functions during DIV1 infection were identified using RNAi. To our surprise, the results showed that the silence of *LvTPI-like* or *LvTPI-Blike1* significantly reduced the survival rate of *L. vannamei*. This result can be attributed to the important role of TPI in both glycolysis and phospholipid biosynthesis, and the silence of *LvTPI-like* or *LvTPI-Blike1* caused the metabolic disorders, affecting the normal life activities of *L. vannamei* (43). It is notable that *LvTPI-Blike1* expression at 48 h post-dsRNA injection was not significantly lower than the control. That may be because the *L. vannamei*, in which *LvTPI-Blike1* was knocked down, died before the detection time point. On these bases, only the survival rates of *LvTPI-like* and *LvTPI-Blike* knock-down *L. vannamei* after DIV1 infection were investigated. It was shown that the silence of *LvTPI-like* and *LvTPI-Blike* did not lead to a significant difference in shrimp survival, but the DIV1 copies were significantly reduced in all the detected tissues of *LvTPI-Blike* knock-down *L. vannamei* and the hemocytes and the muscle of *LvTPI-like* knock-down *L. vannamei* at 48 hpi. Similar results have been reported in the function analysis of immune genes in *L. vannamei*. Shi et al. showed that the cumulative mortality of *L. vannamei* after WSSV infection had no significant difference between laccase knockdown and the control groups, but the WSSV copies were significantly reduced in laccase knock-down *L. vannamei* (52). Similarly, the knockdown *LvTube, LvPelle*, and *LvTAB2* did not affect the mortality of *L. vannamei* caused by WSSV infection, but could slow down the replication of WSSV in the infected *L. vannamei* (53, 54). All these findings were in accordance with the viewpoint that host physiology could be taken over by the virus to support their replication, and the physiological disorder of the host is not conducive to the replication of the virus. In this study, the reduced DIV1 copies may probably be due to the physiological disorder caused by *LvTPI-like* or *LvTPI-Blike* silence. Considering the important role of TPI in metabolism, it can be speculated that a viral-induced Warburg effect could also be induced in DIV1-infected *L. vannamei*, which is similar to the effect of WSSV infection in shrimp (42). In *E. carinicauda* infected with WSSV, glycolysis was affected and TPI was up-regulated, which produced more GAP which became DHAP, which is necessary for the synthesis of phospholipids. The production of phospholipids then affected WSSV replication (51). However, the mechanism of TPI shrimp during DIV1 infection could not be clarified in this study. Further studies are needed. In addition, what could not be ignored was that the DIV1 copies were not significantly different in the hepatopancreas, intestines, and gill of *LvTPI-like* knock-down *L. vannamei* when compared with the control. It may be related to the functional specificity of the gene in different tissues. In any case, there is no doubt about the fact that TPI-like genes play an important role during DIV1 infection in *L. vannamei*.

In conclusion, we determined the LD_50_ values of DIV1-infected *L. vannamei* and found that *TPI-like* genes played an important role during DIV1 infection in *L. vannamei*. The results were helpful to better understand the immune response mechanism of disease resistance in shrimp, which could provide a theoretical basis for the prevention and the control of the viral disease in shrimp culture and be of great significance for promoting the health and the sustainable development of the shrimp industry.

## Data Availability Statement

The datasets presented in this study can be found in online repositories. The names of the repository/repositories and accession number(s) can be found in the article/Supplementary Material.

## Author Contributions

XL, CS, and SZ conceived and designed the experiments. XL, CW, BW, HQ, SH, and PW collected the samples and performed the experiments. XL and SZ analyzed the data as well as wrote the paper. All authors contributed to the article and approved the submitted version.

## Conflict of Interest

PW was employed by Hainan Zhongzheng Aquatic Science and Technology Co., Ltd. The remaining authors declare that the research was conducted in the absence of any commercial or financial relationships that could be construed as a potential conflict of interest.
