# Supplementary material for: Comparative Transcriptome Analysis of Litopenaeus vannamei Reveals That Triosephosphate Isomerase-Like Genes Play an Important Role During Decapod Iridescent Virus 1 Infection
Source: Front Immunol. 2020 Aug 28;11:1904. doi: 10.3389/fimmu.2020.01904 (PMC7485339; doi:10.3389/fimmu.2020.01904)
Supplement: Supplementary file 1 [file Table_1.docx]

**Supplementary data**

**Table S1 Nucleotide sequences of primers were used in this study**

| **Primer names** | **Sequences (5’-3’)** | **Amplification efficiency (%)** |
| --- | --- | --- |
| WSSV-F | CCTCATCTTCCGACTCATCATC |  |
| WSSV-R | CCTCCACCAACCTCATACCA |  |
| IHHNV-F | TCCAACACTTAGTCAAAACCAA |  |
| IHHNV-R | TGTCTGCTACGATGATTATCCA |  |
| DIV1-F | GGGCGGGAGATGGTGTTAGAT |  |
| DIV1-R | TCGTTTCGGTACGAAGATGTA |  |
| qRT-DIV1-F | TCGTTTCGGTACGAAGATGTA | 94.3 |
| qRT-DIV1-R | TTTCACACTTCCTGATAGTCTTCCAT |  |
| Taqman Probe-DIV1 | TCACAGAAAAGATTCCCGAAATGGTAAAAC |  |
| Unigene051281-RT -F | AGGGATAAAGCGGGAACT | 96.8 |
| Unigene051281-RT -R | AGGGAATGTGAGTGGACGT |  |
| Unigene064464-RT-F | ATTTAGACGGATTTCTGGTAGG | 95.7 |
| Unigene064464-RT-R | GGAGGGACTTGTGATGGAA |  |
| Unigene072846-RT-F | CGAGTTCCTGCTCTTCTTGG | 95.2 |
| Unigene072846-RT-R | TTGGCACTGGCTTTAGGA |  |
| Unigene067577-RT-F | ATAACCGTCGCTGCTCCA | 97.5 |
| Unigene067577-RT-R | CACCGCCATTGCTTGTCT |  |
| Unigene027278-RT-F | CGTGGTCTTATGCCTGGTT | 96.9 |
| Unigene027278-RT-R | AGGGCATCTGTCAGGAACTCTT |  |
| Unigene038694-RT-F | TCCAGGTGGTTCGTGAGC | 95.8 |
| Unigene038694-RT-R | CCTCTTCTTCCTGGACGACT |  |
| Unigene068666-RT-F | CCTTCATGGGTGTTCGTG | 98.2 |
| Unigene068666-RT-R | CCTTCATCTTAACATTTAACCC |  |
| Unigene040974-RT-F | CTTTATTTCCTGTGCCAACG | 97.8 |
| Unigene040974-RT-R | GTCCTCGCTTTCCTACGC |  |
| *LvTPI-Like*-RT-F | GAGATGACCAGCAACTACGGC | 96.7 |
| *LvTPI-Like*-RT-R | CTTGCGGTCGTCCGTTGT |  |
| *LvTPI-Blike*-RT-F | TGGCTGCCCTTCCTGCTA | 98.4 |
| *LvTPI-Blike*-RT-R | TTCTCGGCGATGAGTTGG |  |
| *LvTPI-Blike1*-RT-F | AGAGGCGGTGGTTGGCT | 97.8 |
| *LvTPI-Blike1*-RT-R | CTGGCTCGCTGAAAATGGT |  |
| EF1a-F | ATGGGCTGGTGGAAGAAG | 97.2 |
| EF1a-R | CTGAAGGGGAAGACGGAG |  |
| *LvTPI-Like*-F | GAGATTGGCTAATACGCTGAGGAC |  |
| *LvTPI-Like*-R | ATGACGCCCGTGTTGCTC |  |
| *LvTPI-Like*-T7F | GGATCCTAATACGACTCACTATAGGGAGATTGGCTAATACGCTGAGGAC |  |
| *LvTPI-Like*-T7R | GGATCCTAATACGACTCACTATAGGATGACGCCCGTGTTGCTC |  |
| *LvTPI-Blike*-F | GAGGAGGGTCGCACGGA |  |
| *LvTPI- Blike*-R | TAAAAGGGTGAATAACAAAACATACA |  |
| *LvTPI-Blike*-T7F | GGATCCTAATACGACTCACTATAGGGAGGAGGGTCGCACGGA |  |
| *LvTPI-Blike*-T7R | GGATCCTAATACGACTCACTATAGGTAAAAGGGTGAATAACAAAACATACA |  |
| *LvTPI-Blike1*-F | GGCTGTCCATCGTGCTATCTC |  |
| *LvTPI-Blike1*-R | AAGACGCCCGTGTTGCTC |  |
| *LvTPI-Blike1*-T7F | GGATCCTAATACGACTCACTATAGGGGCTGTCCATCGTGCTATCTC |  |
| *LvTPI-Blike1*-T7R | GGATCCTAATACGACTCACTATAGGAAGACGCCCGTGTTGCTC |  |
| EGFP-F | TCAGCGTGTCCGGCGAG |  |
| EGFP-R | TCTTCTGCTTGTCGGCC |  |
| EGFP-T7F | GGATCCTAATACGACTCACTATAGGTCAGCGTGTCCGGCGAG |  |
| EGFP-T7R | GGATCCTAATACGACTCACTATAGGTCTTCTGCTTGTCGGCC |  |
